# Meteorological Table

**Published:** 1812-03

**Authors:** 


### METEOROLOGICAL TABLE.

### *From January 26, to February 26,* 1812.

| | D. | Therm. | | | Barom. | | Hygrom. *Dry.——Damp.* | | | Winds. | Atmos. Variation. |
|---|---|---|---|---|---|---|---|---|---|---|---|
| | 27 | 42 | 48 | 47 | 30 | $29^{9}$ | 30 | — | 25 | SW.. | C ... — ... F .... |
| ○ | 28 | 46 | — | 43 | $29^{8}$ | 7 | 24 | 28 | — | SW... | C ... — .... F .... |
| | 29 | 40 | 42 | 44 | $29^{4}$ | 2 | 20 | 15 | 30 | S... | F .. C ... R .. F.... |
| | 30 | 43 | 46 | 44 | $29^{3}$ | 5 | 26 | — | 22 | S... | F.. — .... R .. — ... |
| | 31 | 40 | 47 | 46 | $29^{8}$ | — | 23 | 21 | 24 | S.. | F .. — ... C ... |
| | 1 | 45 | — | 46 | $29^{7}$ | 6 | 24 | 21 | 24 | SE.. | R .. F .... R .. F .... |
| | 2 | 45 | 41 | — | $29^{6}$ | 5 | 24 | 23 | 26 | SE... | C ... R .. — .. |
| | 3 | 39 | 47 | — | $29^{8}$ | 3 | 26 | 23 | 27 | SE. | F ... C ... R .. C .... |
| | 4 | 47 | 49 | 48 | $29^{4}$ | 5 | 28 | — | 27 | S.. | R .. C ... — ... |
| ◑ | 5 | 48 | 47 | — | $29^{5}$ | 4 | 27 | — | — | S.. | C ... R .. — .... |
| | 6 | 46 | 43 | 45 | $29^{4}$ | 6 | 27 | 25 | 26 | NE.. | Fog ... C .... — ... |
| | 7 | 40 | 45 | 43 | $29^{9}$ | 6 | 23 | 21 | 28 | NW.. | F ... — .... C ... R .. |
| | 8 | 40 | 43 | 41 | $29^{5}$ | 8 | 25 | 20 | 25 | NW.. | F .. C .. R . — . |
| | 9 | 42 | 43 | 42 | $29^{9}$ | — | 24 | 20 | 22 | N.. | Fog .. C ... — ... |
| | 10 | 42 | 44 | 49 | $29^{9}$ | 8 | 20 | 15 | 21 | S..E.. | Fog .. F ... — .... |
| | 11 | 36 | 43 | 42 | $29^{8}$ | 6 | 23 | — | 22 | E.S.. | F .. — ... C ... |
| ● | 12 | 41 | 44 | 47 | $29^{5}$ | 3 | 34 | — | 31 | S. | C ... — ... R .. |
| | 13 | 46 | — | 39 | $29^{4}$ | 7 | 27 | 13 | 22 | NW.. | F .. C .. F .... |
| | 14 | 43 | 46 | 41 | $29^{5}$ | — | 27 | 26 | — | W... | R .. — ... F .... |
| | 15 | 41 | 45 | 43 | $29^{5}$ | 6 | 25 | 18 | 23 | N.. | C ... F ... R .. |
| | 16 | 45 | 48 | 46 | $29^{6}$ | — | 25 | 20 | 27 | W.. | F...C... R... —.. in N |
| | 17 | 49 | 51 | 46 | $29^{6}$ | 4 | 30 | 26 | 27 | W... | R. — .. F .. R .. |
| | 18 | 43 | 47 | 43 | $29^{8}$ | $30^{1}$ | 23 | 11 | 19 | NW.. | R . F .... — .... |
| ◐ | 19 | 44 | 49 | 46 | 30 | — | 24 | 20 | 23 | SW.. | R. F .. C ... F.. |
| | 20 | 42 | 51 | 46 | $29^{9}$ | 8 | 24 | 20 | 23 | SE.S. | F .. — .... — .... |
| | 21 | 47 | 52 | 44 | $29^{7}$ | 5 | 27 | 25 | 26 | SW... | R . F ... R .. — .... |
| | 22 | 44 | 47 | 42 | $29^{4}$ | 5 | 27 | — | 23 | W... | R .... F ... R ... H.., |
| | 23 | 45 | 50 | 44 | $29^{5}$ | — | 27 | 20 | 23 | W... | F .. — ...R... S.. in N |
| | 24 | 36 | 44 | 38 | $29^{4}$ | 9 | 25 | 20 | 23 | NW.. | S .. F ... — .... |
| | 25 | 41 | 43 | 38 | $29^{7}$ | 2 | 23 | 25 | — | SW.. | C .. R .. F .... |
| | 26 | 36 | 42 | — | $29^{3}$ | 6 | 24 | 20 | 22 | NW.. | F ... H .., |

Quantity of rain in this interval 3 inches and $\frac{60}{100}$.

The general character of this period, from Jan. 26th to Feb. 26th, has been unusual mildness: the thermometer has been once, on the 21st, at 52. The wind has blown from a southern point sixteen days; rain on nineteen days; and on two hail and snow.

January 28th. Mild and pleasant. Halo surrounding the Moon.—30th. Gale of wind from S.W. in the evening, with stormy rain.

February 2d. Stormy gale from SE. in the evening.—5th. Misty rain throughout the day; temperature soft and genial. Serene lightning at nine in the evening from the S.—6th. Fog and hazy clouds all day.—7th. Morning brilliantly fine; evaporation rapid; clouds and rain in the afternoon and evening.—8th. The sensible temperature of the air unpleasantly cold.—10th. Notwithstanding wind to S., cold increased, and the night the most severe in the interval.—11th. Fair morning; hoar frost.—12th. Temperature very soft.—14th. Very stormy wind, bearing rather from the W. to the N.; said to have thundered between two and three o'clock, but not heard by the keeper of this register.—21st. Stormy rain with hail in the evening.—22d. Heavy rain in the morning; hail-storm in the afternoon.—24th. Fall of snow in the night of the 23d, covering the streets and houses, but rapidly dissolving.—26th. Hail in the middle of the day.

An opinion prevails very generally, that the state of the atmosphere, with regard to temperature, pressure, and humidity, influences the operations of animal and vegetable systems. With respect to vegetation there can be no doubt; the effects produced on animal functions are not so evident, but still the probability is in favor of health being affected by atmospheric changes. In future we shall endeavor to connect with the actual state of the atmosphere, the actual state of health and disease: interspersing, occasionally, such observations as may arise out of the facts. In the present interval, remarkable for its mild temperature, *Cholera*, a summer and autumnal disease, has not been unfrequent. About the middle of the month, several individuals in the same families, both children and adults, were attacked with vomiting and diarrhœa. *Catarrhal* complaints have been less frequent than is common at this season of the year, but increasing toward the end of the month. Some cases of *Scarlatina* have occurred with anomalies not easily explained. The contagious principle seemed to be either wanting, or at a low standard—the tonsils were inflamed and remarkably tumid in all the cases—the efflorescence on the skin was not present in every instance—œdema of the feet and face succeeded—and in one instance an alarming affection of the head occurred.

*Prince's Street, Cavendish Square, February* 26.

